# Chemical inhibitors of *Candida albicans* hyphal morphogenesis target endocytosis

**DOI:** 10.1038/s41598-017-05741-y

**Published:** 2017-07-18

**Authors:** Hagit Bar-Yosef, Nora Vivanco Gonzalez, Shay Ben-Aroya, Stephen J. Kron, Daniel Kornitzer

**Affiliations:** 1 Department of Molecular Microbiology, B. Rappaport Faculty of Medicine, Technion – I.I.T. and the Rappaport Institute for Research in the Medical Sciences, Haifa, 31096 Israel; 2 Department of Molecular Genetics and Cell Biology, University of Chicago, Chicago, IL 60637 USA; 3 Faculty of Life Sciences, Bar-Ilan University, Ramat-Gan, 52900 Israel

## Abstract

*Candida albicans* is an opportunistic pathogen, typically found as a benign commensal yeast living on skin and mucosa, but poised to invade injured tissue to cause local infections. In debilitated and immunocompromised individuals, *C. albicans* may spread to cause life-threatening systemic infections. Upon contact with serum and at body temperature, *C. albicans* performs a regulated switch to filamentous morphology, characterized by emergence of a germ tube from the yeast cell followed by mold-like growth of branching hyphae. The ability to switch between growth morphologies is an important virulence factor of *C. albicans*. To identify compounds able to inhibit hyphal morphogenesis, we screened libraries of existing drugs for inhibition of the hyphal switch under stringent conditions. Several compounds that specifically inhibited hyphal morphogenesis were identified. Chemogenomic analysis suggested an interaction with the endocytic pathway, which was confirmed by direct measurement of fluid-phase endocytosis in the presence of these compounds. These results suggest that the activity of the endocytic pathway, which is known to be particularly important for hyphal growth, represents an effective target for hyphae-inhibiting drugs.

## Introduction

*Candida albicans* is a commensal organism of the gastrointestinal tract which can cause superficial mucosal membrane infections in immunocompetent and immunocompromised individuals, as well as life-threatening, systemic infection in immunocompromised or debilitated patients^1^. Candidemia accounts for some 9% of nosocomial bloodstream infections^2, 3^, of which 40–70%, depending on the geographic location and specific patient population, are caused by *C. albicans*, and the rest by other *Candida* species^4, 5^. Candidemia can develop into deep-seated candidiasis when the fungus invades internal organs^6^. The mortality rate for invasive candidiasis is between 30% and 40%, a figure that has remained stable for several decades^3, 5, 7, 8^ in spite of the introduction of new classes of antifungals such as the echinocandins^9^.

One of the central and defining characteristics of *C. albicans* is its ability to switch between a yeast form of growth, with rounded cells that disperse after septation, and a hyphal, or mold form, characterized by branching chains of tubular cells without constrictions at the sites of septation^10^. Intermediate patterns, dubbed pseudohyphal forms, are characterized by chains of elongated yeast cells. A variety of environmental stimuli are known to promote the switch to hyphal growth in *C. albicans*: neutral or alkaline pH, carbon starvation, nitrogen starvation, cell density, oxygen concentration, and elevated temperature (>35 °C) (reviewed in refs 11, 12). Incubation in serum at 37 °C is a potent stimulus, and provides the basis for a diagnostic test for *C. albicans* in the clinical laboratory.

Several signal transduction regulators, notably components of the MAPK-^13^ and cAMP/PKA-dependent pathways^14, 15^, serve critical roles in mediating the yeast-to-hyphal switch. A number of transcription factors have been identified that can influence filamentous growth, however only overexpression of CaUme6 can induce true hyphae^16, 17^. Induction of the yeast-to-hyphal switch activates a transcription program characterized by upregulation of genes encoding hyphal cell surface components such as the Hwp1, Ece1 and Als3 proteins^18–20^, as well as the cyclin Hgc1, which is essential for for hyphal morphogenesis^21^ and which is regulated by CaUme6^22^.

It is likely that the hyphal morphology enhances the ability to penetrate the mucous membranes and underlying tissues to enter the bloodstream, a key step en route to candidemia^23^. Hyphal cells are also protected from killing by neutrophils and macrophages^24^, and are necessary for optimal biofilm formation on synthetic substrates^25^. In further support of the importance of hyphal morphogenesis in pathogenicity, *C. albicans* mutants unable to switch from the yeast form to the hyphal form demonstrate significantly reduced virulence in a mouse model of systemic infection^21, 26^. Conversely, studies of strains engineered so that the yeast-to-hyphal switch can be regulated *in vivo*, suggested that hyphal morphogenesis after injection into the bloodstream is essential for virulence^16, 27^. In turn, inhibiting hyphal morphogenesis early in the infection significantly increased survival of the host^28^. Together with the observation that *C. albicans* is found predominantly in hyphal form in tissue samples of candidiasis patients^1^, these observations strongly implicate the yeast-to-hyphal morphogenetic switch in establishing candidemia and in the subsequent tissue invasion. Challenging this view, a genomic screen to identify determinants of hyphal growth and/or virulence revealed only partial overlap, leading to the suggestion that hyphal growth may not be essential for virulence^29^. However, the *in vitro* hyphal growth assay used in the latter study was a poor proxy for *in vivo* behavior and the mouse model of disseminated candidiasis may not fully model a human infection^12^. Furthermore, recent global analysis reaffirmed the link between hyphal morphogenesis and virulence, by showing that among 177 mutant strains tested, attenuation of virulence was significantly correlated with decreased hyphal morphogenesis^30^. Therefore, most investigators continue to recognize a strong link between hyphal morphogenesis and *C. albicans* pathogenicity^31, 32^.

More prevalent than systemic candidiasis are superficial mucosal infections that, although usually less threatening than invasive disease, can impose a significant burden on patients, and can affect both healthy and immunosuppressed individuals. The most common types are oral candidiasis, esophageal candidiasis, and vulvovaginal candidiasis^33^. Importantly, these superficial infections also appear linked to the yeast-to-hyphal transition^34–37^.

Most antifungals, like other antimicrobial agents in clinical use, target essential cellular processes^38^. Screens for novel drugs mainly use inhibition of proliferation as benchmark or, when targeting a specific microbial protein for inhibition, focus on essential targets^39^. Therefore, most antifungals in use or development are growth inhibitory (fungistatic) or lethal (fungicidal)^40^. The drug armamentarium against *Candida spp*. includes three major classes of compounds: the azoles that target ergosterol biosynthesis, polyenes (mainly amphotericin B) that target the fungal membrane, and echinocandins that target cell wall synthesis^41^. Each has limitations, including unpredictable efficacy (all), toxicity (polyenes) and acquired resistance (azoles, and, increasingly, echinocandins^42^). The persistent high levels of mortality with disseminated candidiasis underscore the need for new antifungal therapies.

An alternative paradigm that has emerged in the last decade proposes that microbial infections, and fungal disease in particular, could be combatted by inhibiting virulence mechanisms of the microorganisms, rather than proliferation^43, 44^. The best example to date of a successful, if serendipitous, targeting of a virulence factor is the inhibition of *C. albicans* secreted aspartic proteinases (SAPs). SAPs consist of a large family of enzymes that contribute to mucosal candidiasis^45, 46^. The observation that AIDS patients treated with HIV protease inhibitors showed a reduced incidence of oral candidiasis^47^ has been attributed in part to direct inhibition of *Candida* SAPs^48, 49^. Another example is inhibition of the accumulation of melanin in the *Cryptococcus neoformans* cell wall by the herbicide glyphosate. Although glyphosate has no effect on growth of the fungus *per se*, it enhanced survival of mice experimentally infected with *C. neoformans* ^50^.

Given the importance of the yeast-to-hyphal switch in *C. albicans* virulence, targeting morphogenesis has been proposed as a potential prophylaxis and/or therapy for candidiasis^31, 32, 51^. A number of molecules have been characterized so far that interfere with hyphal morphogenesis. They include agents with poor potential for further development, including lithium, azoles, rapamycin, geldanamycin, histone deacetylase inhibitors, propranolol, actin antagonists, hydroxyurea, nocodazole^51^. Additional reports^52, 53^ describe results of high-content screening for agents that specifically affect the formation of hyphal cells. Their data report a small number of simple small molecules with moderate toxicity at concentrations that impair hyphal growth (1–100 µM). Limited chemical analysis did not reveal high affinity inhibitors based on these agents. In the second study, along with a large number of cytotoxic/cytostatic agents, they reported sixteen drugs and inhibitors that appear to specifically block hyphal growth without impairing cell proliferation. Of the known agents, several kinase and phosphatase inhibitors completely suppressed hyphal growth at ~50 µM. Other agents with a range of known targets in humans (G protein-coupled receptors, calcium homeostasis) had similar effects at 20–100 µM. The effect of these inhibitors was mostly limited to specific hyphal-induction conditions, and they were notably inactive on serum-induced hyphae^54^. More recently, two molecules were identified in screens for biofilm formation inhibitors that also strongly inhibited hyphal morphogenesis, filastatin^55^ and a diazaspiro-decane^56^. The cellular target of these compounds is unknown.

Drug repurposing (repositioning, re-profiling or re-tasking) is an alternative strategy for drug discovery based on finding new uses for existing compounds such as approved drugs. It is estimated that well over 10,000 agents have either been approved for use in the U.S. or elsewhere, or have been examined in Phase II clinical trials^57^. Mining this compound space for possible new uses has the potential to considerably reduce the time and cost of drug development, since the molecules have already well-known safety and pharmacokinetic profiles. Here we describe a screen for compounds from repurposing libraries that inhibit hyphal formation in serum at 37 °C. We identify a chemogenetic interaction of one of these compounds with the endocytic pathway, and show that several hyphal-repressing compounds interfere with fluid-phase endocytosis.

## Results

### Identification of novel hyphal inhibitors

The Microsource Spectrum Collection, NIH Clinical Collections I and II, and Sigma’s LOPAC, over 4000 compounds in all, were screened for inhibitors of hyphal formation as described in Methods. Briefly, an overnight culture of *C. albicans* was diluted in high-glucose DMEM medium with 10% serum and grown in microtiter wells in the presence of compound concentrations ranging from 150 to 0.06 μM. After 3.5 h growth at 37 °C, the cell cultures were fixed with formaldehyde and each well was examined visually with an inverted microscope. While all libraries contained compounds that inhibited growth at 150 μM, only 23 compounds (all found in the NIH Clinical Collection I, but some also found in additional collections) appeared to target filamentation at a concentration of 50 μM or below (Table 1). Prochlorperazine, trifluoperazine, CGS 12066B, disulfiram, and ticlopidine (Supplementary Fig. S1) are compounds that sustained an effect on *C. albicans* morphology at this concentration and were chosen for further analysis based on their availability. Prochlorperazine and trifluoperazine are dopamine receptor antagonists^58^, whereas CGS 12066B is a serotonin-1B receptor agonist^59^. Triflu﻿operazi﻿ne was previously shown to inhibit N-acetylglucosamine-induced hyphal morphogenesis in *C. albicans* ^60^. Disulfiram is an aldehyde dehydrogenase inhibitor that has been shown to exhibit antimicrobial activity along with its first metabolite, diethyldithiocarbamate^61^, which is a cytochrome P450 inhibitor like ticlopidine^62^. Disulfiram was previously identified as a growth inhibitor in *C. albicans*, with its activity depending at least in part on its inhibition of the multidrug resistance (MDR) transporter Cdr1^63^. Prochlorperazine was also identified as an MDR inhibitor that can sensitize *C. albicans* to fluconazole at sub-inhibitory concentrations^64, 65^.

**Table 1:** Novel hyphal induction inhibitors.

Name	Concentration (μM)					
	50	25	12.5	6.2	3.1	1.6
5-Nonyloxytrypta-mine	++/Tx	++/Tx	+/−/Tx	—	—	—
Aripiprazole	++/Tx	+/Tx	+/−/Tx	—	—	—
Benproperine	++/Tx	+/Tx	—	—	—	—
Bifonazole	+/Tx	+/−/Tx	+/−/Tx	−/Tx	—	—
Cerivadtatin Na	+/Tx	—	—	—	—	—
CGS 12066B	++/Tx	++/Tx	+/Tx	+/−/Tx	—	—
Clofazimine	+/Tx	+/−/Tx	—	—	—	—
Diphenylcyclo-propenone	++/Tx	+/Tx	+/−/Tx	—	—	—
Disulfiram	+/Tx	+/−/Tx	—	—	—	—
Ebselen	++/Tx	+/Tx	—	—	—	—
Fluphenazine	+/Tx	—	—	—	—	—
Indatraline	++/Tx	+/Tx	+/−/Tx	—	—	—
Lofepramine	++/Tx	+/Tx	—	—	—	—
Naftopidil	++/Tx	+/Tx	—	—	—	—
Pergolide	++/Tx	+/Tx	+/−/Tx	—	—	—
Prochlorperazine	++/Tx	+/Tx	+/−/Tx	+/−/Tx	—	—
Pterostilbene	++/Tx	+/Tx	—	—	—	—
Stiripentol	++/Tx	+/Tx	+/−/Tx	—	—	—
Tegaserod maleate (Zelnorm)	++/Tx	+/−/Tx	—	—	—	—
Terbinafine	++/Tx	+/Tx	—	—	—	—
Ticlopidine	++/Tx	+/Tx	—	—	—	—
Toremifene	++/Tx	+/−/Tx	—	—	—	—
Trifluoperazine	++/Tx	+/Tx	+/−/Tx	—	—	—

Tx (for Toxic) indicates that visual observation suggested a reduction in growth. ++ stands for yeast cells in colonies or chains, + stands for more yeast than hyphae, +/− stands for more hyphae than yeast, and – stands for hyphae only.

**Figure 1:**
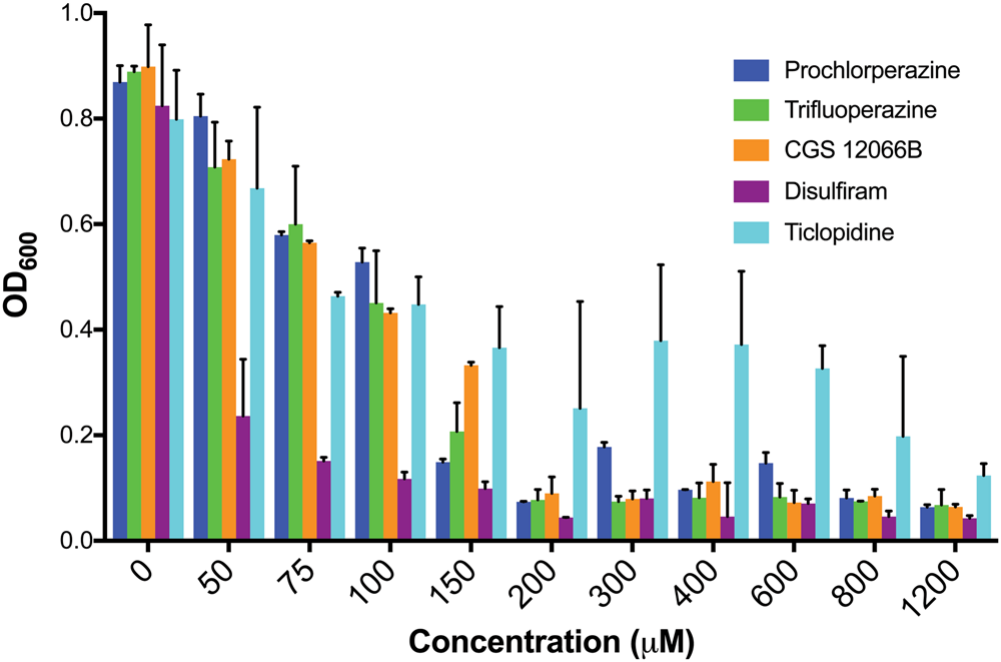
Minimal Inhibitory Concentration of indicated compounds. The effect of five hyphal growth inhibitors was measured on *C. albicans* NCPF 3135 growth in YPD at 30°C for 24 h. The graph indicates the mean ± S.D. of three cultures for each compound and concentration.

Visual observation suggested that the compounds tested often inhibited proliferation in addition to inhibiting hyphal morphogenesis (Table 1). To obtain a more quantitative estimate of the effect on proliferation of the five most active compounds, cells were grown in increasing concentrations of the compounds (Fig. 1). Disulfiram appears to be the most potent inhibitor of growth, with a significant reduction already observed at 50 μM, whereas ticlopidine appeared to be the least active. The ticlopidine effect was however poorly reproducible across different replicates at higher concentrations, possibly indicative of poor solubility in growth medium. Prochlorperazine, trifluoperazine, and CGS 12066B start showing growth effects at 50 μM, with complete growth inhbition above 200 μM. We mostly focused our subsequent analyses on trifluoperazine (TFP) and CGS 12066B.

### Effects of TFP and CGS 12066B on ectopically induced hyphae

In an attempt to identify the step in the hyphal induction pathway that is affected by these compounds, we designed strains that carry a maltose-inducible copy of one of three genes: the cyclin *HGC1* with a C-terminal truncation predicted to activate it, the transcription factor *UME6*, and the MAPKK *STE11* with an N-terminal truncation predicted to activate it. Induction of any of these three genes causes the cells to form true hyphae (Supplementary Fig. S2).

**Figure 2:**
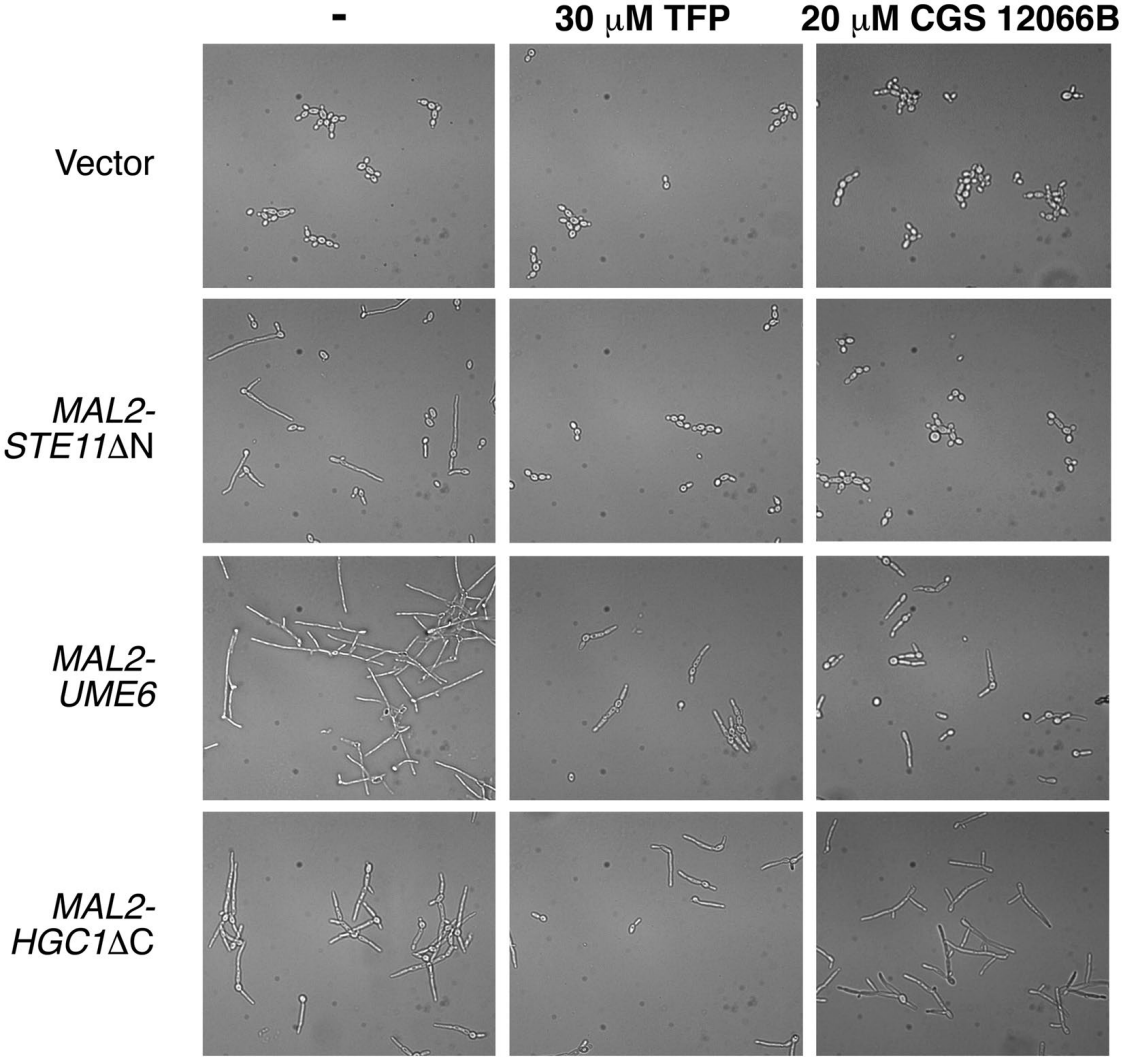
Inhibition of ectopically induced hyphal morphogenesis by TFP and CGS 12066B. *C. albicans* SN148 carrying the vector plasmid KB1018 (KC685) or *MAL2p-STE11*ΔN (KC753), *MAL2p-UME6* (KC763) and *MAL2p-HGC1*ΔC (KC754) were grown overnight in YPD + 2% raffinose, then diluted into medium containing 2% maltose + 30 µM TFP or 20 µM CGS 12066B or no compound, as indicated, and incubated for 5 h at 30 °C. Pictures were taken with a 40X objective equipped with DIC optics.

We first tested which drug concentrations inhibit growth under conditions of maltose induction of these genes. TFP had no effect on growth up to 40 µM, and at 75 µM, growth inhibition was complete (Supplementary Fig. S3). CGS 12066B started displaying a slight growth inhibition at 20 µM, and at 75 µM, growth inhibition was complete (Supplementary Fig. S3). We next tested the effect of sub-inhibitory concentrations of the drugs (30 µM TFP, 20 µM CGS 12066B) on induction of hyphae by the three genes. Both compounds showed similar effects: complete inhibition of morphogenesis induced by Ste11, almost complete inhibition of morphogenesis induced by CaUme6 and limited inhibition of morphogenesis induced by Hgc1 (Fig. 2).

**Figure 3:**
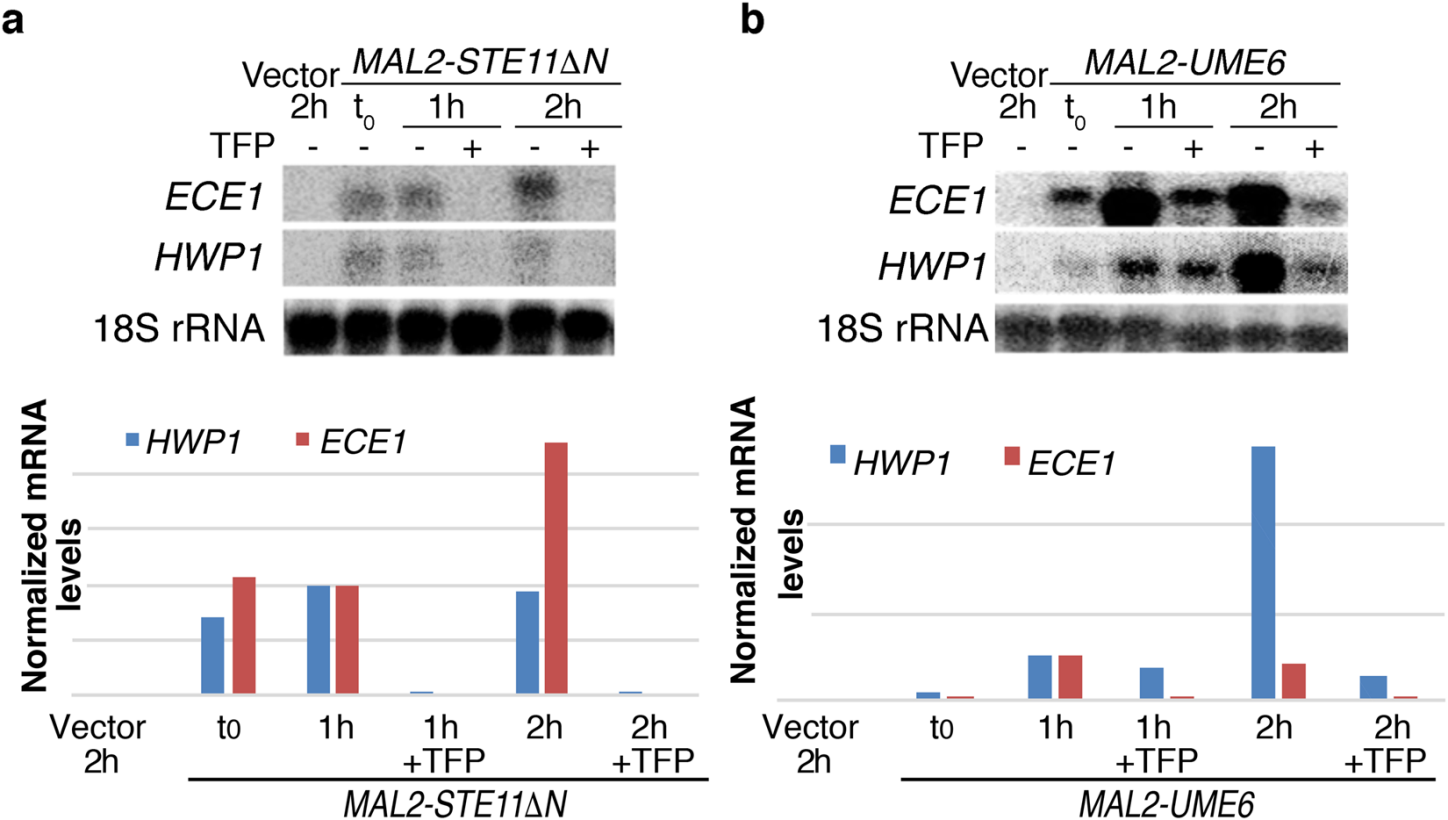
Expression of the hyphal-specific genes *HWP1* and *ECE1* is suppressed by TFP. *C. albicans* SN148 cells were ectopically induced to produce hyphae by induction of *STE11ΔN* (KC753) (**a**) or *UME6* (KC763) (**b**) under the *MAL2* promoter. Cells were grown overnight in 2% raffinose and shifted to 2% maltose for the indicated amounts of time in the presence or absence of 10 µM TFP. *ECE1* and *HWP1* transcript levels were normalized to 18 S rRNA. All readings were then normalized to the 1 h induction point ( = 1).

To test whether the inhibition of morphogenesis was accompanied by an inhibition of the expression of hyphal-specific genes (HSGs), we tested expression of the HSGs *HWP1* and *ECE1* after induction of Ca*UME6* or Ca*STE11*, with or without 10 µM TFP. As shown in Fig. 3, while induction of both Ca*UME6* and Ca*STE11* led to the induction of *HWP1* and *ECE1*, presence of TFP considerably reduced this induction, consistent with the reduction in hyphal morphogenesis.

### Effect of TFP on biofilm formation

**Figure 4:**
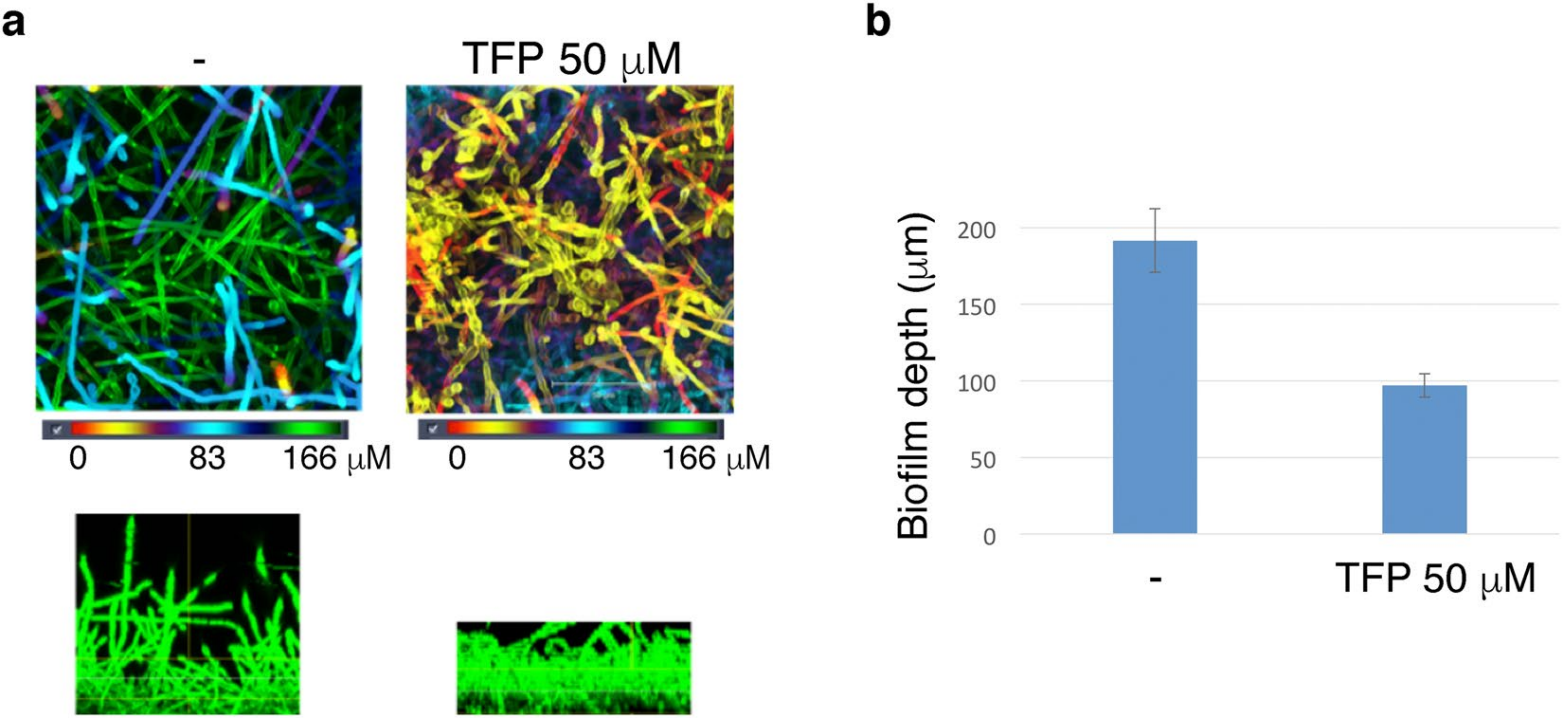
Biofilm formation is partially inhibited upon exposure to Trifluoperazine. Medical silicone sheeting was incubated with *C. albicans* (KC403) under standard biofilm-inducing conditions (46 hr, 37 °C), with or without 50 µM Trifluoperazine. (**a**) Top: CSLM biofilm depth views in which red represents cells closest to the silicone substrate and green represents cells farthest from the silicone substrate. Bottom: CSLM side views. (**b**) Average depth of biofilm with and without TFP on 4 silicone sheets each. Error bars indicate the standard deviations.

Biofilm formation on medical devices by many microorganisms, including *C. albicans*, constitutes a growing problem in the clinic. Optimal biofilms include a large proportion of hyphal cells that are thought to act as scaffolding^25^. In order to test whether TFP could interfere with biofilm growth, we performed a biofilm formation test on silicone squares in a 24-well plate as described in Methods. Treated silicone squares were incubated for 48 hours at 37°C, with shaking at 150 rpm, with or without 50 µM TFP. Biofilms were visualized by confocal laser scanning microscopy (CLSM), with a DAPI filter. A dramatic difference in the depth was detected on the silicone squares incubated with TFP: in these cultures, biofilm depth was reduced on average by 50% (Fig. 4).

### Identification of potential TFP targets in *S. cerevisiae*

**Figure 5:**
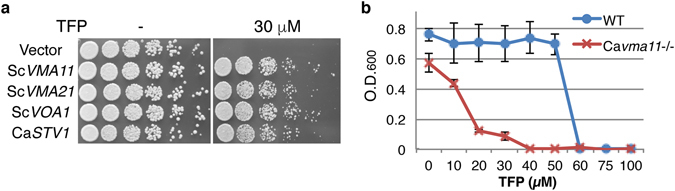
(**a**) Identification of *S. cerevisiae* and *C. albicans* genes that rescue Trifluoperazine toxicity at high copy. Serial dilutions of *S. cerevisiae* cells carrying the indicated genes on a 2 micron plasmid were plated on YPD or YPD + 30 µM TFP plates and incubated for 2 days at 30 °C. (**b**) MIC test of TFP on *C. albicans vma11* ^*−/−*^(KC280) vs. *VMA11* ^*+/+*^ (CAF3-1). The cells were grown in YPD overnight at 37°C in the presence of TFP at the indicated concentrations. The graph indicates the mean ± S.D. of three cultures.

The facile genetic manipulation of the baker’s yeast, *Saccharomyces cerevisiae*, especially when contrasted with *C. albicans*, enables the application of genetic methods to identify potential drug targets. However, in order to be able to utilize *S. cerevisiae* as model organism for identifying candidate TFP and CGS 12066B targets, we first determined whether these compounds affect *S. cerevisiae* growth at all. We found that growth inhibition did occur with both compounds, with *S. cerevisiae* exhibiting a similar or higher sensitivity to CGS 12066B and TFP as *C. albicans* (Supplementary Fig. S4). After confirming that TFP inhibits *S. cerevisiae* growth at 30 µM on plates (Supplementary Fig. S5), we performed a high-copy TFP sensitivity suppression screen, using both a *S. cerevisiae* and a *C. albicans* 2 µ genomic library in BY4741. Candidate plasmids were sequenced, retransformed and re-tested by serial dilution plating on 30 µM TFP plates (Fig. 5A). The *S. cerevisiae* genes identified were *VMA11*, *VMA21* and *VOA1*, and the *C. albicans* gene that consistently rescued TFP toxicity was *STV1*, all genes associated with the vacuolar ATPase: Vma11 and Stv1 are subunits of the V_0_ (transmembrane) subcomplex^66^, and Voa1 and Vma21 are proteins involved in assembly of the V-ATPase in the endoplasmic reticulum^67, 68^.

Since the high-copy suppression screen identified components of the vacuolar ATPase, we asked whether a VMA deletion in *C. albicans* would affect TFP sensitivity. As shown in Fig. 5B, a homozygote *vma11* ^*−/−*^ deletion mutant is significantly more sensitive to TFP than the wild-type strain. In addition, since vacuolar ATPase mutants had been shown to be detective in hyphal morphogenesis^69–71^, we also tested the phenotype of the *vma11* ^*−/−*^ mutant. As shown in Supplementary Fig. S6, this mutant is unable to form hyphae in standard hyphae-induction conditions, confirming the requirement for an active vacuolar ATPase for hyphal morphogenesis.

**Figure 6:**
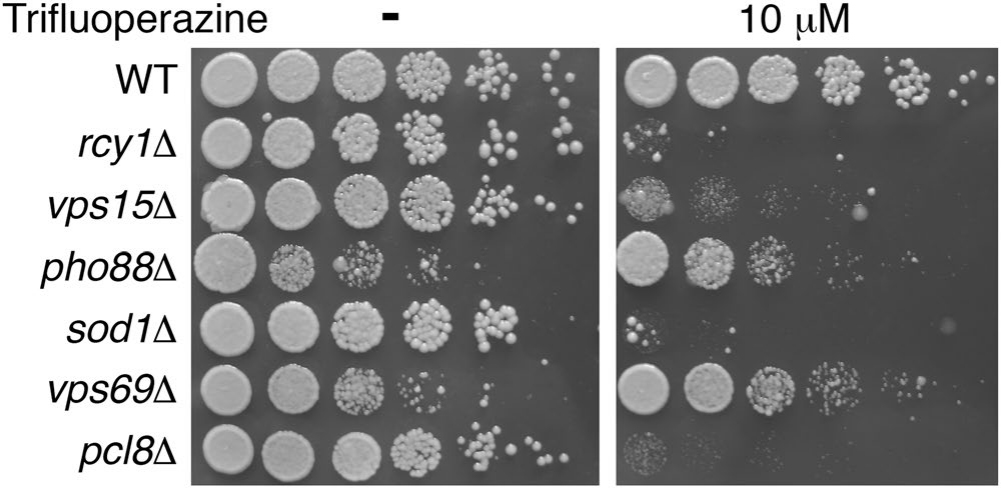
Enhanced sensitivity to Trifluoperazine of *S. cerevisiae* mutants. Serial dilutions of the indicated mutants were plated on YPD or YPD+10 µM TFP plates and incubated for 2 days at 30 °C. Whereas *rcy1∆*, *vps15∆*, *sod1∆* and *pcl8∆* exhibit strongly reduced growth on TFP only, *pho88∆* and *vps69∆* exhibit non-specific reduced growth in the absence of the compound as well.

To complement the TFP high-copy toxicity suppression screen, we next carried out a screen for TFP hypersensitivity using an arrayed *S. cerevisiae* gene deletion library. Most non-essential genes, about 4500, are represented in this library (the EUROSCARF library). The deletion collection was printed with a robotic arrayer on plates containing YPD and YPD+10 µM TFP (a concentration at which the wild-type strain BY4741 grows normally), and the spots were compared for size. Strains that seemed hypersensitive to TFP on the array plates were collected for further analysis: serial dilutions of individual strains were spotted on 10 µM TFP plates. As shown in Fig. 6, deletion of 4 genes were found to confer TFP hypersensitivity: *RCY1*, *VPS15*, *SOD1*, and *PCL8*, while two other candidate deletions did not show enhanced sensitivity upon re-plating. Rcy1 and Vps15 are particularly interesting, as they are proteins involved in endocytic recycling of membrane proteins, and in targeting of proteins to the vacuole, respectively^72, 73^. Interestingly, two independent yeast high-throughput chemogenomic screens also identified an interaction of TFP with endocytic and vesicle transport. For example, among the top interacting genes in these screens were the clathrin *CLC1* and the clathrin-interacting protein *SWA2* ^74^, and the vesicle transport genes *VPS35* and *SNF7* (as well as *RCY1*)^75^. Together with the identification of the vacuolar ATPase in the high-copy suppression screen, which is responsible for the maintenance of acidic pH in the endocytic pathway, we started to discern a pattern of genes involved in protein trafficking and endocytosis as the target of TFP.

### Effect of TFP, CGS 12066B and prochlorperazine on fluid-phase endocytosis

**Figure 7:**
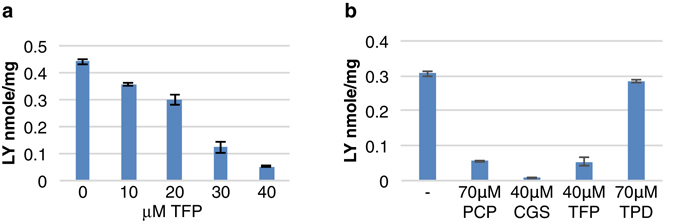
Effect of tested drugs on fluid-phase endocytosis. (**a**) Lucifer Yellow uptake was measured in *C. albicans* (SN148) cells after growth for 2.5 h at 37 °C with different concentrations of trifluoperazine. The graph indicates the mean ± S.D. of three samples of the same culture. (**b**) Lucifer Yellow uptake was measured as in **a**, without or with prochlorperazine (PCP), CGS 12066B, trifluoperazine and ticlopidine (TPD). The graph indicates the mean ± S.D. of three cultures.

The chemogenetic interaction of TFP with endocytosis prompted us to assay the effect of TFP on fluid-phase endocytosis. Fluid-phase endocytosis can be assayed by measuring incorporation of the membrane-impermeable fluorophore Lucifer Yellow (LY)^76^. As shown in Fig. 7a, TFP significantly inhibits LY uptake at concentrations that do not inhibit growth but that were found to inhibit hyphal morphogenesis. We further tested additional drugs identified in our screen for inhibitors of hyphal morphogenesis (Fig. 7b). Whereas ticlopidine had no effect on fluid-phase endocytosis as measured by LY uptake, CGS 12066B and prochlorperazine profoundly inhibited endocytosis at concentrations that inhibit hyphal morphogenesis, similar to TFP. This suggests that the effect on hyphal morphogenesis of these three drugs may be linked to their effect on endocytosis.

## Discussion

The current antifungal armamentarium is limited by the small number of available drug classes, by toxic side-effects of existing drugs, and by the emergence of increasing drug resistance among fungal pathogens. Targeting virulence rather than proliferation has been suggested as a way to increase the number of available drug targets, and to potentially reduce selective pressures leading to resistance^43, 77^. In *C. albicans*, the morphogenetic switch from yeast to hyphae constitutes a virulence factor that could serve as target for treatment of both systemic and mucosal infections^31, 32^. Here, we screened several libraries of relatively well-characterized drugs (“drug repurposing” screen) in order to identify inhibitors of hyphal morphogenesis.

The three most active specific inhibitors of hyphal morphogenesis were two closely related piperazine-type phenothiazines, prochlorperazine and trifluoperazine, and another piperazine, CGS 12066B. While these drugs inhibited hyphal formation at intermediate concentrations, they inhibited cell proliferation at higher concentrations. TFP is an extensively investigated drug that was shown to inhibit several mammalian cell targets, including the dopamine receptor^58^, the calmodulin-dependent Ca^++^-ATPase^78^, and a sodium channel^79^. In an attempt to identify the relevant TFP target in *C. albicans*, we took advantage of the sensitivity of the related baker’s yeast to TFP, to perform chemogenomic analysis. An initial screen for high-copy suppressors with either a *S. cerevisiae* or a *C. albicans* library yielded several subunits of the vacuolar ATPase. Another vacuolar ATPase gene had been previously isolated as suppressor of TFP toxicity in yeast^80^. The vacuolar ATPase acidifies the vacuole and the endosome, suggesting an interaction of TFP with the exocytic and endocytic patway. We next looked for hypersensitive strains in the yeast deletion collection. Of the four mutants identified, two pointed to the endocytic pathway; in addition, two large-scale chemogenomic screens that had been performed in the interval^74, 75^ also suggested an interaction of TFP with proteins involved in endocytosis and vesicular transport. We confirmed that the endocytic pathway is a target of the drugs identfied in our screen, by showing that TFP, prochlorperazine and CGS 12066B all inhibit fluid-phase endocytosis in *C. albicans*.

Genetic analysis has already indicated that vacuolar ATPase is essential for hyphal morphogenesis^69–71^. Likewise, a role for endocytosis in *C. albicans* hyphal growth had been suggested by the requirement for proteins involved in fluid phase endocytosis such as Pan1^81^, myosin type I Myo5^82^, the Wiscott-Aldrich Syndrome Protein homolog Wal1^83^, the verprolin Vrp1^84^, and the BAR domain proteins Rvs161 and Rvs167^85^. The likely explanation for a requirement for endocytosis in hyphal morphogenesis is the need to recycle membranes as well as membrane proteins deposited by vesicle exocytosis at the tip of the extending hypha (reviewed in ref. 86). Our work provides further support for this interaction by linking pharmacological inhibition of endocytosis to inhibition of hyphal morphogenesis.

A surprising observation was that the inhibition of hyphal morphogenesis was accompanied by a decrease in hyphal-specific gene expression. Together with the effect on cells ectopically expressing CaUme6, this led us initially to suspect that TFP and CGS 12066B target CaUme6 or another part of the gene expression machinery. However, in view of the chemogenomic interactions detected with TFP, combined with their effect on fluid-phase endocytosis, we favor the model that these drugs affect hyphal extension via their effect on the endocytic pathway. This model does however not explain why HSG expression is affected by TFP. The possibility remains that in addition to its effect on endocytosis, TFP also affects substrates in the hyphal induction pathway. However it seems more likely that the reduction of hyphal elongation *via* reduction in endocytosis causes decreased HSG expression. This would imply that the hyphal morphogenetic apparatus feeds back into the hyphal gene expression program. While the mechanism of this feedback is unknown, it fits with the notion that not only external signals, but also interference with cellular physiology such as e.g. cell cycle progression, can induce HSG expression and hyphal morphogenesis^87^.

Our intention in assaying a repurposing drug library was to identify hyphal suppressors among molecules of known safety. However, the concentrations required for even partial hyphal inhibition (>10 µM) appear to be above the safe serum levels of these drugs^88^. Nonetheless, our results suggest that the endocytic pathway and the vacuolar ATPase might constitute useful targets for pharmacological inhibition of hyphal morphogenesis. This raises the prospect of identifying molecules that target fungal endocytosis with high affinity as potential inhibitors of *C. albicans* virulence.

## Materials and Methods

### Strains and plasmids

Inhibition of the yeast-to-hyphae switch was tested in the *C. albicans* NCPF 3153 strain (London Mycological Reference Laboratory)^89^. Ectopic hyphal induction was carried out in strain SN148^90^ carrying plasmids KB2147 (*MAL2*-Ca*UME6*)^17^, KB2264, KB2265, or the plasmid vector BES119^91^. Biofilm formation was assayed in KC403, i.e. strain CAF3–1 transformed with plasmid BES119. KC280 is a deletion of both alleles of Ca*VMA11* ^92^.

The yeast (*S. cerevisiae*) deletion mutant collection consists of approximately 4700 strains, each carrying the strain BY4741 auxotrophic markers (MATa *ura3∆0 leu2Δ0 his3Δ1 met15Δ0*), and a gene deletion mutation linked to a KanMX marker, which confers resistance to the antibiotic geneticin^93^.

Plasmid KB2264 contains Ca*STE11* between positions 1402–2473 cloned PstI-ApaI in KB1610^94^, enabling to express a 6xMyc-tagged N-terminal truncation of Ste11 under the *MAL2* promoter. The N-terminal truncation of Ste11 causes it to be hyperactive compared to the full-length gene. KB2265 contains the *HGC1* open reading frame between positions 1–1206, cloned PstI-ApaI in KB1610 ^93^, enabling to express a 6xMyc-tagged C-terminal truncation of Hgc1 under the *MAL2* promoter. The 2 micron plasmid *C. albicans and S. cerevisiae* genomic libraries used were obtained respectively from Haoping Liu (UC Irvine, CA, USA)^95^ and from Phil Hieter (UBC, Vancouver, Canada)^96^.

### Compound collections

We used the Microsource Spectrum Collection, NIH Clinical Collections I and II, and Sigma’s LOPAC. The Microsource Spectrum Collection is a set of diverse 2,320 compounds, including drugs, natural products, and bioactive compounds. The NIH Clinical Collections are composed of 769 drugs that are currently in phases 1 to 3 of clinical trials. The LOPAC collection from Sigma encompasses 1,280 drugs and small molecule modulators. The commonly used antifungal clotrimazole was used as a control.

### Visual screen of yeast-to-hyphae switch inhibition

Prior to each screen, a *C. albicans* yeast colony was inoculated in 5 mL of yeast peptone medium plus 2% glucose (YPD) and grown overnight at 30 °C with shaking. The next day 3 μL of each compound was manually distributed from their 5 mM stocks in DMSO (stored at −20°C) into the top row of wells from a Costar 3370 microplate (untreated, flat bottom, clear polystyrene, 96-wells, and with low evaporation lid). Immediately before the screen, cells were washed twice with ultra pure water and diluted 1:1000 in either fresh YPD (control, noninducing medium) or Gibco’s high glucose DMEM supplemented with L-glutamine, phenol red, 100 units/mL penicillin and 100 µg/mL streptomycin, and 10% fetal calf serum (hyphae-inducing medium). Subsequently, 100 μL of hyphae inducing medium was added to each well of the microplate. A 1:3 or 1:2 serial dilution was then carried out to obtain concentrations ranging from 150 to 0.06 μM along each microplate column in a final volume of 67 μL in each well. For the yeast morphology control, cells were diluted into non-inducing medium and kept at 30 °C, while for the hyphal morphology control, cells were not treated with a compound. Unless otherwise specified, the microplates were incubated at 37°C for 3.5 hrs.

For a morphological analysis of *C. albicans* NCPF 3153 strain in the microplate, an Axiovert 40 CFL microscope (Zeiss) was used. After the 3.5 hr time point, cells were fixed with 2% paraformaldehyde (PFA) and two phase-contrast images of each well were taken from each of the two replicates. Observations were noted as – (hyphae),+/− (minority of yeast cells),+(majority of yeast cells) and ++ (only yeast cells) (see Supplementary Fig. S7). Tx (for Toxic) indicates that visual observation suggested a reduction in growth.

The hit rate was 12/2320 for the Microsource collection, 10/1280 for the Lopac collection, 23/446 for the NIH clinical collection I and 0/281 for the NIH clinical collection II. However, due to duplications in the collections, all the hits from the two first collections were also found among the hits of the NIH collection.

### Determination of the minimum inhibitory concentration (MIC)

The growth of *C. albicans* NCPF 3153 strain was analyzed using a microplate assay. Each compound was tested at final concentrations of 1200, 800, 600, 400, 300, 200, 150, 100, 75, 50, and 0 μM. From an overnight culture grown in 5 mL of YPD at 30°C with shaking, cells were diluted to 2000 cells/mL in fresh YPD, of which 150 was added to each Costar 3370 microplate well. The microplate was set to incubate overnight (24 hrs) at 30°C with shaking on a rotary platform. Prior to a reading with the Tecan Safire II at 600 nm (OD600), cells in each well were resuspended using a multichannel pipettor. Two replicates were conducted for this assay.

### Biofilm formation assay

Sterilized silicone squares were placed in a 24-well plate (1 per well), with 1 mL of RPMI 1640+HEPES+NaHCO_3_+2% glucose to each well and the plate placed overnight at 37 °C with 150 rpm shaking. An overnight culture of *C. albicans* strain KC403 was inoculated in YPD. The next day, cell were resuspended in RPMI, added to silicone squares and incubated at 37°C with 150 rpm shaking for 2 hours in order for the cells to adhere. Next, the silicone squares were carefully washed and transferred to a new 24 well plate, each well was filled with 1 mL of fresh RPMI medium. At this stage, 50 µM TFP was added to the medium of some of the wells. The plate was incubated for 48 hours at 37°C, with shaking at 150 rpm. After incubation, samples were fixed with formaldehyde. The cells were stained with calcofluor white and viewed by confocal laser scanning microscopy (CLSM), with a DAPI filter. The pictures were analyzed by the program IMARIS 8.1.

### Fluid-phase endocytosis assay

Lucifer Yellow (LY) uptake was used as a measure of fluid-phase endocytosis^76^. Overnight cultures were diluted in the appropriate medium and grown 2–3 h to an OD_600_ of 0.1. For each assay, 10 ml of the culture was spun down (3500 g, 5 min) and resuspended in 0.9 ml of fresh medium. 0.1 ml of Lucifer Yellow CH (LY; Sigma), 40 mg/ml in water, was added and the cultures were incubated another 1 h while shaking. The cells were then spun down, resuspended in 1 ml ice-cold stop buffer (50 mM Succinate-NaOH, pH 7.5, 100 mM NaCl, 10 mM NaN_3_, 10 mM MgCl_2_), and spun down and resuspended again for a total of 6 times. The final pellet was resuspended in 1 ml lysis buffer (50 mM Tris-Cl Ph 7.5 m 10 mM 2-mercaptoethanol, 2000 U lyticase (Sigma) / ml) and incubated 15’ at 37 °C. LY fluorescence was measured at 426 nm excitation and 550 nm emission using a Tecan Infinite M200 PRO spectrofluorometer. Fluorescence was calibrated with free LY in the same buffer. To normalize LY uptake to cell amount, the protein amount was measured by the Bradford method (Bio-Rad protein assay dye reagent), using 0.2 ml of the same extract used for LY quantitation. Bovine serum albumin (Sigma) was used for calibration of the protein assay.

### Data availability

All data generated or analysed during this study are included in this published article (and its Supplementary Information files).

## Electronic supplementary material

Supplementary information

## References

[1] Richardson, M. D. & Warnock, D. W. *Fungal infection - diagnosis and management*, (Blackwell Sciences Ltd., Oxford, 1997).

[2] Pfaller MA, Diekema DJ (2007). Epidemiology of invasive candidiasis: a persistent public health problem. Clin Microbiol Rev.

[3] Pfaller MA, Diekema DJ (2010). Epidemiology of invasive mycoses in North America. Critical reviews in microbiology.

[4] Falagas ME, Roussos N, Vardakas KZ (2010). Relative frequency of albicans and the various non-albicans *Candida spp* among candidemia isolates from inpatients in various parts of the world: a systematic review. International journal of infectious diseases: IJID: official publication of the International Society for Infectious Diseases.

[5] Pfaller M (2012). Epidemiology and outcomes of candidemia in 3648 patients: data from the Prospective Antifungal Therapy (PATH Alliance(R)) registry, 2004-2008. Diagn Microbiol Infect Dis.

[6] Kullberg BJ, Arendrup MC (2015). Invasive Candidiasis. N Engl J Med.

[7] Gudlaugsson O (2003). Attributable mortality of nosocomial candidemia, revisited. Clinical infectious diseases: an official publication of the Infectious Diseases Society of America.

[8] Hassan I, Powell G, Sidhu M, Hart WM, Denning DW (2009). Excess mortality, length of stay and cost attributable to candidaemia. The Journal of infection.

[9] Mora-Duarte J (2002). Comparison of caspofungin and amphotericin B for invasive candidiasis. The New England journal of medicine.

[10] Sudbery P, Gow N, Berman J (2004). The distinct morphogenic states of *Candida albicans*. Trends Microbiol.

[11] Cottier F, Muhlschlegel FA (2009). Sensing the environment: response of *Candida albicans* to the X factor. FEMS Microbiol Lett.

[12] Sudbery PE (2011). Growth of *Candida albicans* hyphae. Nature reviews Microbiology.

[13] Csank C (1998). Roles of the *Candida albicans* mitogen-activated protein kinase homolog, Cek1p, in hyphal development and systemic candidiasis. Infect Immun.

[14] Rocha CR (2001). Signaling through adenylyl cyclase is essential for hyphal growth and virulence in the pathogenic fungus *Candida albicans*. Mol Biol Cell.

[15] Cloutier M (2003). The two isoforms of the cAMP-dependent protein kinase catalytic subunit are involved in the control of dimorphism in the human fungal pathogen *Candida albicans*. Fungal Genet Biol.

[16] Carlisle PL (2009). Expression levels of a filament-specific transcriptional regulator are sufficient to determine *Candida albicans* morphology and virulence. Proc Natl Acad Sci USA.

[17] Mendelsohn S, Pinsky M, Weissman Z, Kornitzer D (2017). Regulation of the *Candida albicans* hyphainducing transcription factor Ume6 by the CDK1 cyclins Cln3 and Hgc1. mSphere.

[18] Kadosh D, Johnson AD (2005). Induction of the *Candida albicans* filamentous growth program by relief of transcriptional repression: a genome-wide analysis. Mol Biol Cell.

[19] Lane S, Birse C, Zhou S, Matson R, Liu H (2001). DNA array studies demonstrate convergent regulation of virulence factors by Cph1, Cph2, and Efg1 in *Candida albicans*. J Biol Chem.

[20] Nantel A (2002). Transcription profiling of *Candida albicans* cells undergoing the yeast-to-hyphal transition. Mol Biol Cell.

[21] Zheng X, Wang Y (2004). Hgc1, a novel hypha-specific G1 cyclin-related protein regulates *Candida albicans* hyphal morphogenesis. EMBO J.

[22] Carlisle PL, Kadosh D (2010). *Candida albicans* Ume6, a filament-specific transcriptional regulator, directs hyphal growth via a pathway involving Hgc1 cyclin-related protein. Eukaryot Cell.

[23] Koh AY, Kohler JR, Coggshall KT, Van Rooijen N, Pier GB (2008). Mucosal damage and neutropenia are required for *Candida albicans* dissemination. PLoS pathogens.

[24] Lorenz MC, Bender JA, Fink GR (2004). Transcriptional response of *Candida albicans* upon internalization by macrophages. Eukaryot Cell.

[25] Nobile CJ, Johnson AD (2015). *Candida albicans* Biofilms and Human Disease. Annu Rev Microbiol.

[26] Lo H-J (1997). Nonfilamentous *C. albicans* mutants are avirulent. Cell.

[27] Saville SP, Lazzell AL, Monteagudo C, Lopez-Ribot JL (2003). Engineered control of cell morphology *in vivo* reveals distinct roles for yeast and filamentous forms of *Candida albicans* during infection. Eukaryotic cell.

[28] Saville SP (2006). Inhibition of filamentation can be used to treat disseminated candidiasis. Antimicrobial agents and chemotherapy.

[29] Noble SM, French S, Kohn LA, Chen V, Johnson AD (2010). Systematic screens of a *Candida albicans* homozygous deletion library decouple morphogenetic switching and pathogenicity. Nat Genet.

[30] O’Meara TR (2015). Global analysis of fungal morphology exposes mechanisms of host cell escape. Nat Commun.

[31] Jacobsen ID (2012). *Candida albicans* dimorphism as a therapeutic target. Expert Rev Anti Infect Ther.

[32] Vila T (2017). Targeting *Candida albicans* filamentation for antifungal drug development. Virulence.

[33] Ruhnke, M. Skin and mucous membrane infections. In *Candida and candidiasis* (ed. Calderone, R.A.) 307–325 (ASM press, Washington, 2002).

[34] Sobel JD, Muller G, Buckley HR (1984). Critical role of germ tube formation in the pathogenesis of candidal vaginitis. Infection and immunity.

[35] Martin MV, Craig GT, Lamb DJ (1984). An investigation of the role of true hypha production in the pathogenesis of experimental oral candidosis. Sabouraudia.

[36] Hisajima T (2008). Invasion process of *Candida albicans* to tongue surface in early stages of experimental murine oral candidiasis. Medical mycology: official publication of the International Society for Human and Animal Mycology.

[37] Peters BM (2014). Fungal morphogenetic pathways are required for the hallmark inflammatory response during *Candida albicans* vaginitis. Infect Immun.

[38] Murray, P. R., Rosenthal, K. S. & Pfaller, M. A. *Medical microbiology*, (Mosby Elsevier, Philadelphia, PA, 2009).

[39] Roemer T (2011). Confronting the challenges of natural product-based antifungal discovery. Chemistry & biology.

[40] Ostrosky-Zeichner L, Casadevall A, Galgiani JN, Odds FC, Rex JH (2010). An insight into the antifungal pipeline: selected new molecules and beyond. Nature reviews. Drug discovery.

[41] Denning DW, Hope WW (2010). Therapy for fungal diseases: opportunities and priorities. Trends in microbiology.

[42] Katiyar S, Pfaller M, Edlind T (2006). *Candida albicans* and Candida glabrata clinical isolates exhibiting reduced echinocandin susceptibility. Antimicrobial agents and chemotherapy.

[43] Clatworthy AE, Pierson E, Hung DT (2007). Targeting virulence: a new paradigm for antimicrobial therapy. Nat Chem Biol.

[44] Gauwerky K, Borelli C, Korting HC (2009). Targeting virulence: a new paradigm for antifungals. Drug discovery today.

[45] Naglik, J. R., Challacombe, S. J. & Hube, B. *Candida albicans* secreted aspartyl proteinases in virulence and pathogenesis. *Microbiology and molecular biology reviews: MMBR* **67**, 400–28, table of contents (2003). 10.1128/MMBR.67.3.400-428.2003 PMC193873 12966142

[46] Naglik JR (2008). Quantitative expression of the *Candida albicans* secreted aspartyl proteinase gene family in human oral and vaginal candidiasis. Microbiology.

[47] Hoegl L, Thoma-Greber E, Rocken M, Korting HC (1998). HIV protease inhibitors influence the prevalence of oral candidosis in HIV-infected patients: a 2-year study. Mycoses.

[48] Korting HC (1999). Effects of the human immunodeficiency virus (HIV) proteinase inhibitors saquinavir and indinavir on *in vitro* activities of secreted aspartyl proteinases of *Candida albicans* isolates from HIV-infected patients. Antimicrobial agents and chemotherapy.

[49] Cassone A (1999). *In vitro* and *in vivo* anticandidal activity of human immunodeficiency virus protease inhibitors. J Infect Dis.

[50] Nosanchuk JD, Ovalle R, Casadevall A (2001). Glyphosate inhibits melanization of *Cryptococcus neoformans* and prolongs survival of mice after systemic infection. The Journal of infectious diseases.

[51] Shareck J, Belhumeur P (2011). Modulation of morphogenesis in *Candida albicans* by various small molecules. Eukaryotic cell.

[52] Toenjes KA (2005). Small-molecule inhibitors of the budded-to-hyphal-form transition in the pathogenic yeast *Candida albicans*. Antimicrobial agents and chemotherapy.

[53] Toenjes KA, Stark BC, Brooks KM, Johnson DI (2009). Inhibitors of cellular signalling are cytotoxic or block the budded-to-hyphal transition in the pathogenic yeast *Candida albicans*. Journal of medical microbiology.

[54] Midkiff J, Borochoff-Porte N, White D, Johnson DI (2011). Small Molecule Inhibitors of the *Candida albicans* Budded-to-Hyphal Transition Act through Multiple Signaling Pathways. PloS one.

[55] Fazly A (2013). Chemical screening identifies filastatin, a small molecule inhibitor of *Candida albicans* adhesion, morphogenesis, and pathogenesis. Proc Natl Acad Sci USA.

[56] Pierce, C. G. *et al*. A Novel Small Molecule Inhibitor of *Candida albicans* Biofilm Formation, Filamentation and Virulence with Low Potential for the Development of Resistance. *NPJ Biofilms Microbiomes* **1** (2015). 10.1038/npjbiofilms.2015.12 PMC4681527 26691764

[57] Chong CR, Sullivan DJ (2007). New uses for old drugs. Nature.

[58] Feinberg AP, Snyder SH (1975). Phenothiazine drugs: structure-activity relationships explained by a conformation that mimics dopamine. Proc Natl Acad Sci USA.

[59] Neale RF (1987). Biochemical and pharmacological characterization of CGS 12066B, a selective serotonin-1B agonist. Eur J Pharmacol.

[60] Gupta Roy, B. & Datta, A. A calmodulin inhibitor blocks morphogenesis in *Candida albicans*. *FEMS Microbiol Lett* **41**, 327–329 (1987).

[61] Horita Y (2012). Antitubercular activity of disulfiram, an antialcoholism drug, against multidrug- and extensively drug-resistant Mycobacterium tuberculosis isolates. Antimicrob Agents Chemother.

[62] Kot M, Daniel WA (2009). Effect of diethyldithiocarbamate (DDC) and ticlopidine on CYP1A2 activity and caffeine metabolism: an *in vitro* comparative study with human cDNA-expressed CYP1A2 and liver microsomes. Pharmacol Rep.

[63] Shukla S, Sauna ZE, Prasad R, Ambudkar SV (2004). Disulfiram is a potent modulator of multidrug transporter Cdr1p of *Candida albicans*. Biochem Biophys Res Commun.

[64] Bulatova NR, Darwish RM (2008). Effect of chemosensitizers on minimum inhibitory concentrations of fluconazole in *Candida albicans*. Med Princ Pract.

[65] Pajak B, Molnar J, Engi H, Orzechowski A (2005). Preliminary studies on phenothiazine-mediated reversal of multidrug resistance in mouse lymphoma and COLO 320 cells. In Vivo.

[66] Graham LA, Flannery AR, Stevens TH (2003). Structure and assembly of the yeast V-ATPase. J Bioenerg Biomembr.

[67] Ryan M, Graham LA, Stevens TH (2008). Voa1p functions in V-ATPase assembly in the yeast endoplasmic reticulum. Mol Biol Cell.

[68] Hill KJ, Stevens TH (1994). Vma21p is a yeast membrane protein retained in the endoplasmic reticulum by a di-lysine motif and is required for the assembly of the vacuolar H(+)-ATPase complex. Mol Biol Cell.

[69] Jia C (2014). Role of TFP1 in vacuolar acidification, oxidative stress and filamentous development in *Candida albicans*. Fungal Genet Biol.

[70] Poltermann S (2005). The putative vacuolar ATPase subunit Vma7p of *Candida albicans* is involved in vacuole acidification, hyphal development and virulence. Microbiology.

[71] Rane HS (2013). *Candida albicans VMA3* is necessary for V-ATPase assembly and function and contributes to secretion and filamentation. Eukaryot Cell.

[72] Wiederkehr A, Avaro S, Prescianotto-Baschong C, Haguenauer-Tsapis R, Riezman H (2000). The F-box protein Rcy1p is involved in endocytic membrane traffic and recycling out of an early endosome in *Saccharomyces cerevisiae*. J Cell Biol.

[73] Herman PK, Stack JH, DeModena JA, Emr SD (1991). A novel protein kinase homolog essential for protein sorting to the yeast lysosome-like vacuole. Cell.

[74] Hoepfner D (2014). High-resolution chemical dissection of a model eukaryote reveals targets, pathways and gene functions. Microbiol Res.

[75] Lee AY (2014). Mapping the cellular response to small molecules using chemogenomic fitness signatures. Science.

[76] Dulic V (1991). Yeast endocytosis assays. Methods in enzymology.

[77] Allen RC, Popat R, Diggle SP, Brown SP (2014). Targeting virulence: can we make evolution-proof drugs?. Nat Rev Microbiol.

[78] Hinds TR, Raess BU, Vincenzi FF (1981). Plasma membrane Ca2+ transport: antagonism by several potential inhibitors. J Membr Biol.

[79] Sheets PL (2006). Inhibition of Nav1.7 and Nav1.4 sodium channels by trifluoperazine involves the local anesthetic receptor. J Neurophysiol.

[80] Shih CK, Kwong J, Montalvo E, Neff N (1990). Expression of a proteolipid gene from a high-copy-number plasmid confers trifluoperazine resistance to *Saccharomyces cerevisiae*. Mol Cell Biol.

[81] Martin R (2007). Functional analysis of *Candida albicans* genes whose *Saccharomyces cerevisiae* homologues are involved in endocytosis. Yeast.

[82] Oberholzer U, Marcil A, Leberer E, Thomas DY, Whiteway M (2002). Myosin I is required for hypha formation in *Candida albicans*. Eukaryot Cell.

[83] Walther A, Wendland J (2004). Polarized hyphal growth in *Candida albicans* requires the Wiskott-Aldrich Syndrome protein homolog Wal1p. Eukaryot Cell.

[84] Borth N (2010). *Candida albicans* Vrp1 is required for polarized morphogenesis and interacts with Wal1 and Myo5. Microbiology.

[85] Douglas LM, Martin SW, Konopka JB (2009). BAR domain proteins Rvs161 and Rvs167 contribute to *Candida albicans* endocytosis, morphogenesis, and virulence. Infect Immun.

[86] Shaw BD, Chung DW, Wang CL, Quintanilla LA, Upadhyay S (2011). A role for endocytic recycling in hyphal growth. Fungal Biol.

[87] Woolford CA (2016). Bypass of *Candida albicans* Filamentation/Biofilm Regulators through Diminished Expression of Protein Kinase Cak1. PLoS Genet.

[88] Schulz M, Schmoldt A (2003). Therapeutic and toxic blood concentrations of more than 800 drugs and other xenobiotics. Pharmazie.

[89] Gow NA, Gooday GW (1982). Growth kinetics and morphology of colonies of the filamentous form of *Candida albicans*. J Gen Microbiol.

[90] Noble M (2005). Exploiting structural principles to design cyclin-dependent kinase inhibitors. Biochim Biophys Acta.

[91] Feng Q, Summers E, Guo B, Fink G (1999). Ras signaling is required for serum-induced hyphal differentiation in *Candida albicans*. J Bacteriol.

[92] Weissman Z, Shemer R, Conibear E, Kornitzer D (2008). An endocytic mechanism for haemoglobin-iron acquisition in *Candida albicans*. Mol Microbiol.

[93] Giaever G (2002). Functional profiling of the *Saccharomyces cerevisiae* genome. Nature.

[94] Atir-Lande A, Gildor T, Kornitzer D (2005). Role for the SCF(CDC4) ubiquitin ligase in *Candida albicans* morphogenesis. Mol. Biol. Cell.

[95] Liu H, Kohler J, Fink GR (1994). Suppression of hyphal formation in *Candida albicans* by mutation of a *STE12* homolog. Science.

[96] Connelly C, Hieter P (1996). Budding yeast *SKP1* encodes an evolutionarily conserved kinetochore protein required for cell cycle progression. Cell.

