# Supplementary material for: Chemical inhibitors of Candida albicans hyphal morphogenesis target endocytosis
Source: Sci Rep. 2017 Jul 18;7:5692. doi: 10.1038/s41598-017-05741-y (PMC5515890; doi:10.1038/s41598-017-05741-y)
Supplement: Supplementary file 1 — Supplementary information [file 41598_2017_5741_MOESM1_ESM.pdf]

SUPPLEMENTARY FIGURES

for

Chemical inhibitors of *Candida albicans* hyphal morphogenesis target endocytosis

Hagit Bar Yosef[1#], Nora Vivanco Gonzalez[2#], Shay Ben Aroya[3], Stephen J. Kron*[2] and Daniel Kornitzer*[1]

[1]Department of Molecular Microbiology, B. Rappaport Faculty of Medicine, Technion – I.I.T. and the Rappaport Institute for Research in the Medical Sciences, Haifa 31096, Israel; [2]Department of Molecular Genetics and Cell Biology, University of Chicago, Chicago IL 60637, USA; and [3]Faculty of Life Sciences, Bar-Ilan University, Ramat-Gan 52900, Israel

[#] Equal contribution

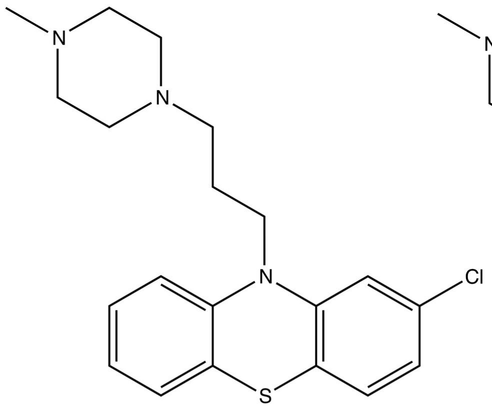

Prochlorperazine

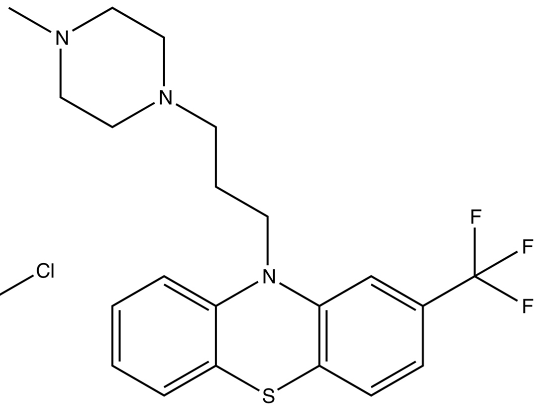

Trifluoperazine

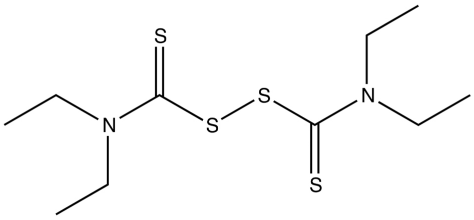

Disulfiram

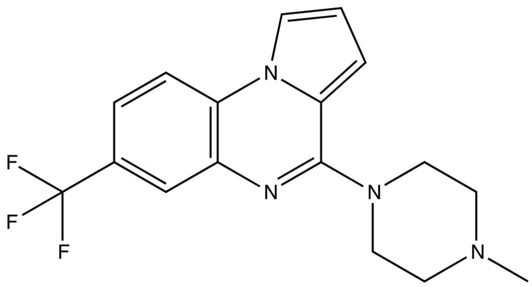

CGS 12066B

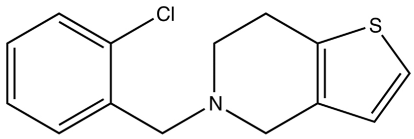

Ticlopidine

Fig S1. Chemical structure of the five most active compounds suppressing hyphal morphogenesis.

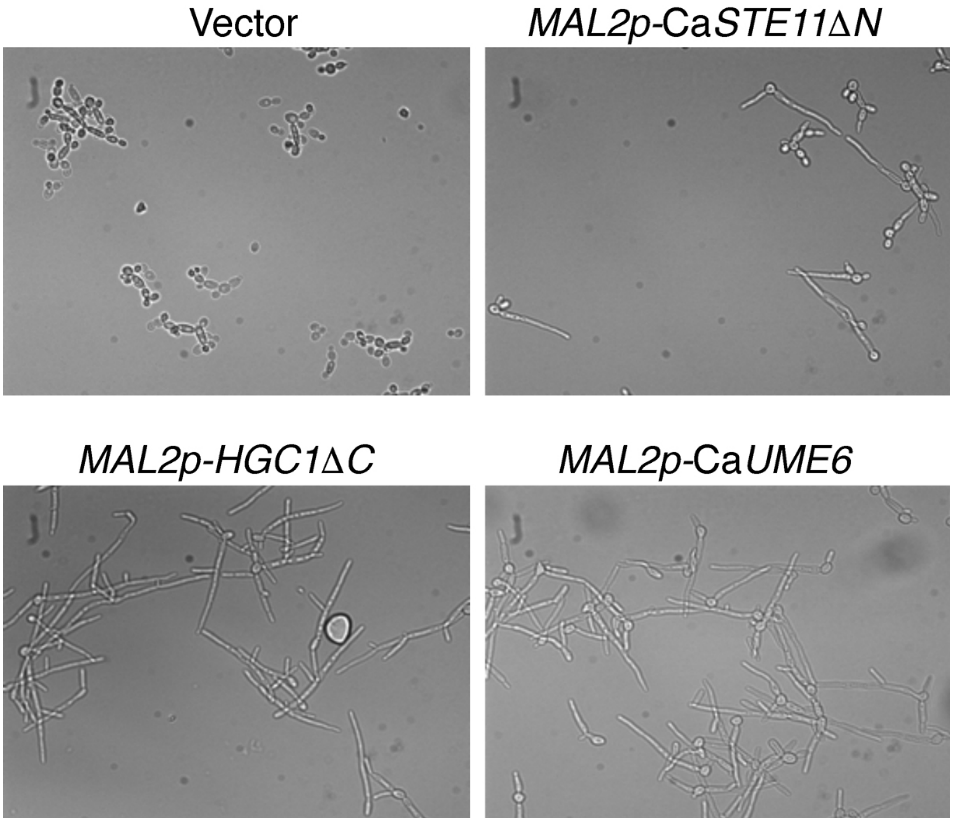

Figure S2. *C. albicans* SN148 carrying the vector plasmid BES119 (KC685) or *MAL2p-STE11*ΔN (KC753), *MAL2p-UME6* (KC763) and *MAL2p-HGC1* ΔC (KC754) were grown overnight in YPD + 2% raffinose, then diluted into medium containing 2% maltose and photographed after growth for 5 hours. Pictures were taken with a 40X objective equipped with DIC optics.

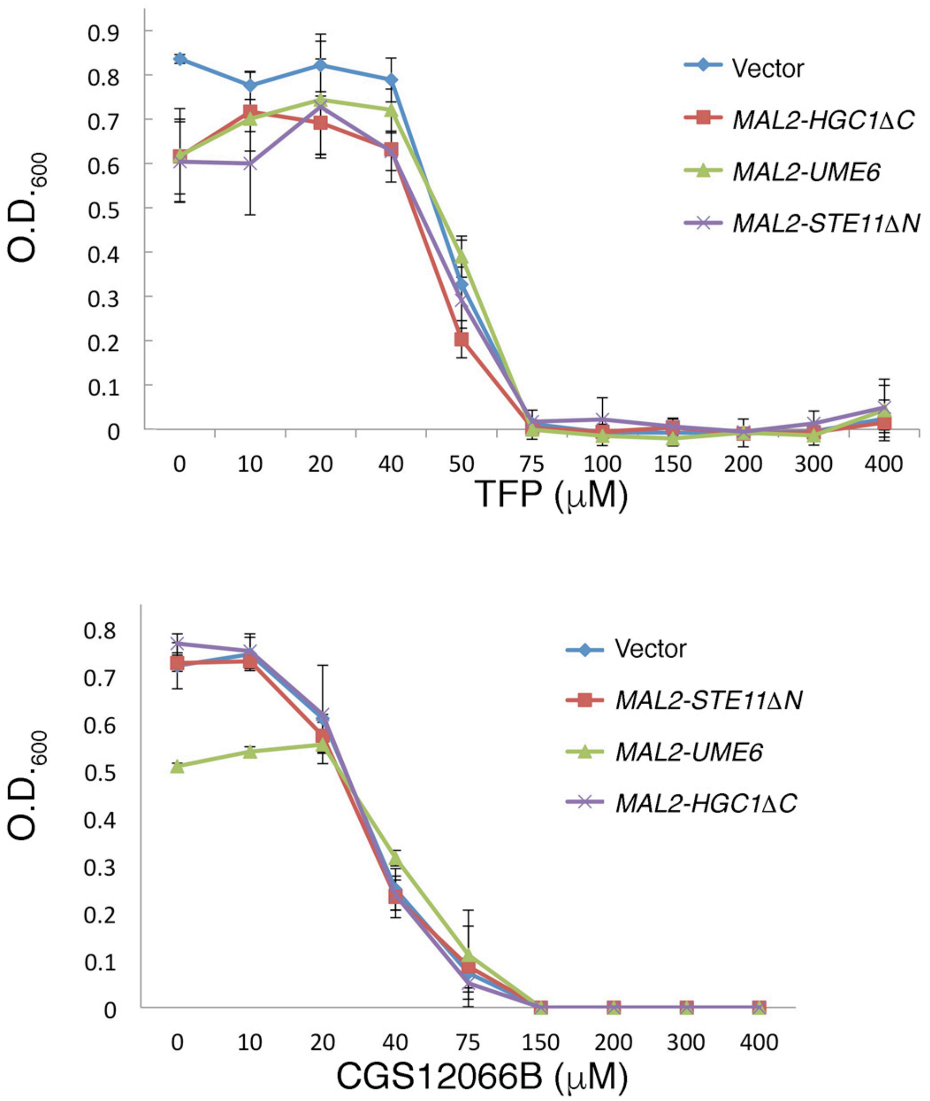

Fig S3. *C. albicans* SN78 carrying the vector plasmid BES119 (KC685) or *MAL2p-STE11*ΔN (KC753), *MAL2p-UME6* (KC763) and *MAL2p-HGC1* ΔC (KC754) were grown overnight in medium with maltose at 30˚C in the presence of TFP or CGS 12066B at increasing concentration, as indicated. The results are the mean ± S.D. of the optical density of three cultures each.

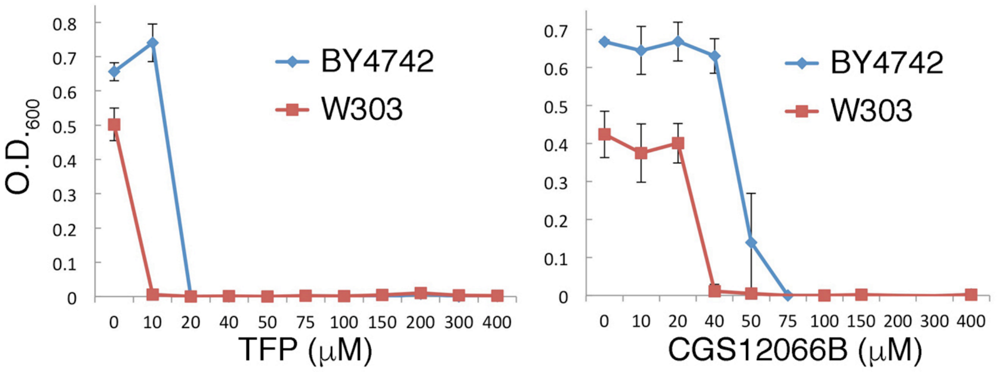

Fig. S4. MIC test of Trifluoperazine and CGS 12066B on *S. cerevisiae*. The indicated strains of *S. cerevisiae* were grown overnight in YPD medium at 30˚C in the presence of TFP or CGS 12066B at increasing concentrations. The results are the mean ± S.D. of the optical density of three cultures each.

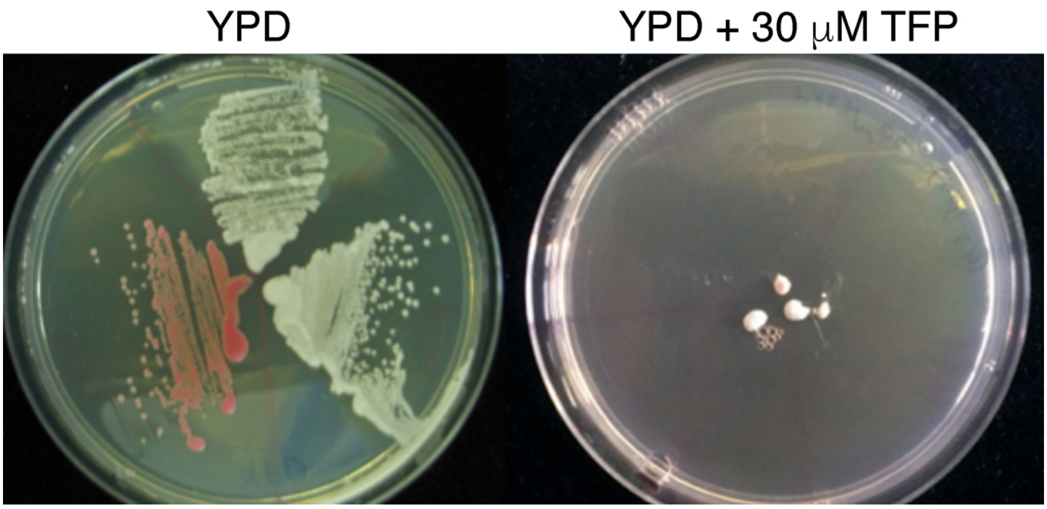

Fig. S5. TFP toxicity on plates. Three strains of *S. cerevisiae* (Top: BY4741, right: BY4742, left: W303) were streaked on YPD or YPD + 30μM TFP as indicated, and grown for 2 days at 30˚C.

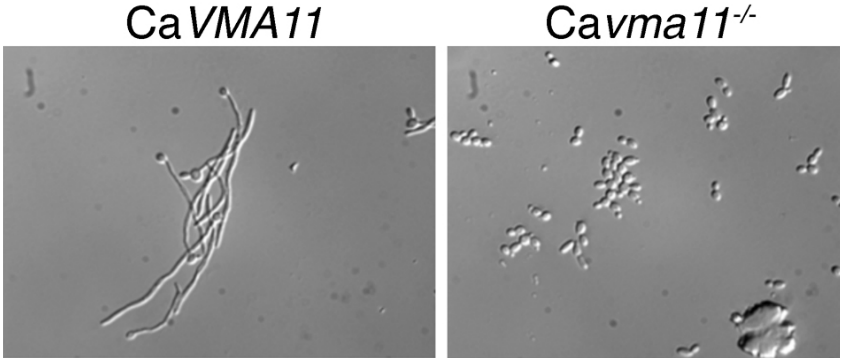

Fig. S6. Effect Ca*VMA11* deletion on hyphal morphology. Strains of *C. albicans* wild type (CAF3-1) and a *VMA11* knockout strain (KC280) were grown in YPD + 10% FCS for 5 hours at 37°C. Pictures were taken with a 40X objective.

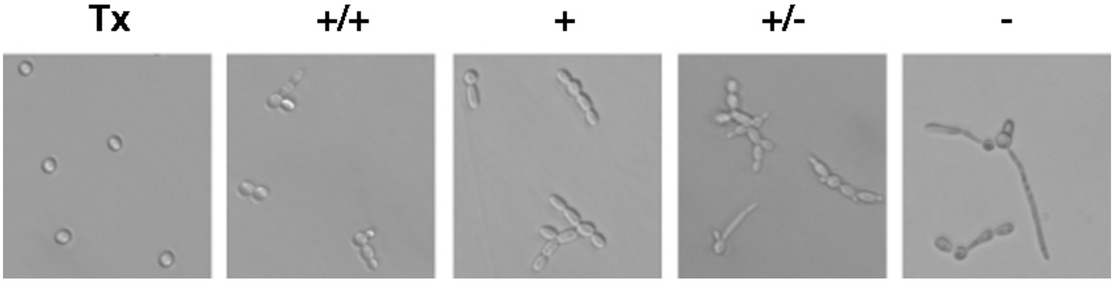

Fig. S7. Examples of the assignment of hyphal morphologies by visual inspection as used in the screen for hyphal inhibitors.
